# Effect of TiB_2_ Content on Microstructure and Mechanical Properties of TiB_2_/Al-Zn-Mg-Cu Composites with High Zn Content

**DOI:** 10.3390/ma18225191

**Published:** 2025-11-15

**Authors:** Wenchao Sun, Zhilei Xiang, Jihao Li, Zian Yang, Yang Han, Ziyong Chen

**Affiliations:** Faculty of Materials and Manufacturing, Beijing University of Technology, Beijing 100124, China; wenchaos0206@163.com (W.S.);

**Keywords:** TiB_2_/Al-Zn-Mg-Cu composites, microstructure, interfaces, peak aging, mechanical properties

## Abstract

The addition of reinforcement particles can considerably improve the mechanical properties of 7xxx series aluminum alloy. In this work, the effects of TiB_2_ reinforcement particles on the microstructure, mechanical properties, strengthening mechanisms, and aging precipitation of TiB_2_/Al-Zn-Mg-Cu composites were systematically investigated using scanning electron microscopy (SEM), transmission electron microscopy (TEM), and tensile testing machine. The results indicate that when the TiB_2_ content is 1 wt.%, the composite achieves a tensile strength of 831 MPa while maintaining an elongation of 6.7%, meeting the research objectives of this experiment. When the aging heat treatment temperature is set at 120 °C, the peak aging time is shortened to 20 h. The interfacial phase composed of solute elements preferentially nucleates near the TiB_2_ particles during the cooling process. With the increase in TiB_2_ content, clustering in localized regions slows down the diffusion rate of interfacial phases into the matrix, thereby increasing the required duration of the solution treatment. Excellent interfacial relationships exist between TiB_2_ particles and both the aluminum matrix and the MgZn_2_ phase. It is also found that with the increase in TiB_2_ content, the aging-hardness response of TiB_2_/Al-Zn-Mg-Cu composites is accelerated and the work hardening rate is reduced. In addition, a multi-component strengthening model for the yield strength of the composite was established based on various strengthening mechanisms, including second-phase strengthening, dislocation strengthening, age-precipitation strengthening, and fine-grain strengthening. The results indicate that age-precipitation strengthening and dislocation strengthening are the most significant contributors to strength in the composite.

## 1. Introduction

7xxx series aluminum alloys are widely used in military equipment, marine, and transportation fields due to their high specific stiffness, high specific strength, ease of processing and forming, excellent toughness, and corrosion resistance [1,2,3]. In response to the era of rapid development, researchers have consistently conducted extensive research on 7xxx series aluminum alloys with high Zn content [4,5]. Experimental results indicate that adding a high content of Zn element to Al-Zn-Mg-Cu aluminum alloys can accelerate the age-hardening response rate, thereby significantly enhancing the hardness and tensile properties of the alloy [6]. In addition to increasing the content of Zn element [7,8], the incorporation of high-modulus ceramic particles (such as SiC [9,10], Al_2_O_3_ [11] and TiB_2_ [12,13,14]) into aluminum alloys has opened up new research avenues for significantly enhancing their elastic modulus and strength.

Low density, flexible processing, and good strength have attracted attention to composites based on Al alloys [15]. Reinforcing AA6061 aluminum alloys with materials such as silicon carbide (SiC) significantly improves their mechanical properties, including hardness, wear resistance, and tensile strength [16]. Due to its high modulus, excellent chemical stability, and good compatibility with aluminum alloy matrices, TiB_2_ has attracted widespread attention in the study of aluminum matrix composites [17]. In numerous research reported, the preparation methods for aluminum matrix composites containing TiB_2_ reinforcement particles primarily follow two principles: the in situ method and the ix situ method. Among these, the in situ method is highly favored due to its advantages of low cost, small particle size, clean interfaces, and strong bonding with the matrix. However, this method also faces challenges: the high interfacial energy between nano/micro-scale particles, significant density differences between the melt and particles [13], and melt convection cause the generated TiB_2_ particles to agglomerate easily at grain boundaries [18]. This results in a much higher volume fraction of particles in the grain boundary regions compared to the grain interiors [19,20]. During thermo-mechanical processing, due to the particle stimulate nucleation (PSN) effect, larger particles (>1 μm) can stimulate dynamic recrystallization [21,22]. Smaller particles (<1 μm) can hinder the recovery process by pinning dislocations [21]. Thus, the existence of TiB_2_ particles has a profound impact on the grain boundary structure and the distribution of grain misorientation. Additionally, the increased alloying complexity in Al-Zn-Mg-Cu systems promotes the formation of coarse secondary phase networks at the grain boundaries during fabrication. This is because the electronegativity values of Al and Zn atoms are extremely close, resulting in negligible electron transfer between them. Consequently, the Al-Zn binary system does not form stable intermetallic compounds [23]. Geng et al. found that part of the second phase was attached to the growth of sub-micron TiB_2_ particle clusters, and the diffusion of elements was inhibited during heat treatment, resulting in a large number of coarse phase residues [20]. Residual coarse secondary phases can induce stress concentration during material processing, severely impairing the mechanical properties of the alloy and adversely affecting its service quality and lifespan. Hot deformation processes including hot extrusion, forging, hot rolling and friction stir processing are common and effective means for treating particle-reinforced composites. This thermomechanical processing can significantly refine particle clusters and coarse secondary phases, leading to improved microstructural homogeneity and a reduction in defects. In addition, Ceschini et al. [11]. found that the forging process can significantly reduce the grain size of Al_2_O_3_/AA6061 composites, which is mainly attributed to dynamic recrystallization. In addition, Chung et al. [24]. found that extrusion deformation can effectively refine the grains of Al-Zn-Mg-Cu alloys, which they attributed to the occurrence of dynamic recrystallization. The intermetallic compounds are broken during the extrusion process, and the solid solution and aging precipitation reactions are more rapid during the subsequent heat treatment process. The extrusion process can also effectively reduce the number of pores and particle clusters. In addition, Tham et al. [25]. studied the extruded SiC/Al composites, and also found that the hot extrusion process reduced the small holes caused by SiC particles and made the SiC particles evenly distributed, thereby improving the mechanical properties. Wu et al. [26] and Nitish Raja et al. [27] employed ultrasonic stirring treatment to achieve a uniform dispersion of particles and fine, equiaxed recrystallized grains, thereby enhancing both strength and ductility. Most of these studies optimize the microstructure of composites by deformation process to greatly improve the mechanical properties. However, these experimental methods can only play a supporting role in eliminating particle clusters and improving particle distribution, and the effect is limited. Moreover, there are few studies on the optimal TiB_2_ addition in Al-Zn-Mg-Cu alloys with high Zn content (12 wt.%). Composition optimization plays an important role in the development of Al-Zn-Mg-Cu alloys. Furthermore, the content and size of the particles also have a certain effect on the aging precipitation behavior of the Al-Zn-Mg-Cu alloys [28,29]. Previous studies have usually focused on micron-sized particles. Studies have found that the addition of reinforcement particles leads to an earlier onset of age-hardening in composites compared to conventional alloys [29,30], which was attributed to the geometrically necessary dislocations (GND) formed by different thermal expansion coefficients and the plastic deformation zone of the matrix [28,29,30,31]. The effect of nano-sized particles on aging is rarely studied. Aging precipitation has a profound impact on the microstructure and strength of the alloy. Elucidating the role of TiB_2_ particles in the aging process is helpful to better understand the phase transformation and strengthening mechanism of 7xxx series alloy.

## 2. Materials and Methods

All the experiments in this study were completed in Beijing University of Technology. In this research work, the designed composites had TiB_2_ contents of 0 wt.%, 1 wt.%, and 3 wt.%, respectively. Alloy A served as the matrix alloy without TiB_2_ reinforcement particles, Alloy B contained 1 wt.% TiB_2_, and Alloy C contained 3 wt.% TiB_2_. The micro-nano TiB_2_ particles used in this experiment are Al/TiB_2_ seed materials prepared by oxide method using aluminum powder, titanium powder, titanium oxide, and boric acid as raw materials [32]. After extensive preliminary experiments, the final determined contents of Zn, Mg, and Cu elements in the alloy were established as 12 wt.%, 2.2 wt.%, and 1.7 wt.%, respectively. In the process of alloy melting, Zn and Mg produce a small amount of burning loss. Table 1 shows the actual chemical composition of the three alloys obtained by icp detection, which is less different from the design value.

The first step is to prepare TiB_2_/Al-Zn-Mg-Cu-Zr composite ingots by gravity casting. The prepared composite ingots were homogenized in a heat treatment furnace at 455 °C (time: A: 35 h, B: 37 h, C: 40 h). The ingot was extruded into a plate with a thickness of 6 mm and a width of 60 mm at an extrusion ratio of 17:1, and the extrusion temperature was set to 410 °C. The specific heat treatment system is that the extrusion plate is first subjected to solid solution treatment in a heat treatment furnace at 475 °C, and the solid solution time of different alloys is different (a: 1.5 h; b: 2 h; c: 2.5 h). The samples were then transferred into a water-filled tank within 5 s and quenched to room temperature. Subsequently, a single-step long-term aging treatment was carried out in a heat treatment furnace at 120 °C. The time required to reach peak aging differed among the three alloys (a: 24 h; b: 20 h; c: 18 h). The microstructure of the composites was analyzed using a D8 Advance X-ray diffractometer (XRD, Bruker AXS, Karlsruhe, Germany), a scanning electron microscope (SEM, QUANTA FEG 650, FEI Company, HO, USA), and a transmission electron microscope (TEM, JEOL 2100, JEOL Ltd., Tokyo, Japan). XRD sample preparation process: 10 × 10 × 5 mm sheet-shaped samples were cut from both the as-extruded and aged alloy materials. These samples were then ground successively on 80, 400, 1000, and 3000 grit sandpaper to achieve a smooth surface, followed by mechanical polishing. The scanning electron microscope (SEM) is equipped with ancillary facilities including an Energy Dispersive Spectrometer (EDS, FEI Company, HO, USA) and an Electron Backscatter Diffractometer (EBSD, FEI Company, HO, USA). For EBSD sample preparation, mechanical polishing was first conducted, followed by electrolytic polishing using a mixed solution of 30 wt.% HNO_3_ and 70 wt.% CH_3_OH as the electrolyte. For TEM sample preparation, the specimens were mechanically thinned to below 70 μm and then cut into 3 mm diameter discs. At 248 K, a double-jet electrolytic polishing instrument, utilizing a mixed solution of 30 wt.% HNO_3_ and 70 wt.% CH_3_OH as the electrolyte, was employed to prepare electron-transparent specimens with thicknesses less than 40 μm. The high-resolution TEM (HRTEM) images captured by the transmission electron microscope were ultimately analyzed and processed using GMS-3 software (Gatan, Inc. USA). The hardness test is carried out at room temperature using a Vickers hardness tester (Laizhou Laihua Testing Instrument Factory, YanTai City, China), and 10 points are randomly selected from each sample for testing. Tensile tests were conducted at room temperature using a CMT 5205 GL uniaxial tensile testing machine (SanSi test, HangZhou City China), with a crosshead displacement rate of 0.9 mm/min. The tensile specimens were standard flat coupons machined along the extrusion direction of the composite material. A minimum of three tensile tests were performed for each alloy to ensure statistical reliability. Aztec-Crystal software (Oxford Instruments, Oxford, United Kingdom) was used to analyze the EBSD test data. The low-angle grain boundary is defined as the grain boundary orientation difference in the range of 2–15°, and the high-angle grain boundary is defined as the grain boundary with a grain boundary orientation difference greater than 15°. The effect of TiB_2_ particles on the precipitation kinetics was calculated by mathematical equations. In addition, a multi-mode strengthening model was established according to various strengthening mechanisms in the composites.

## 3. Results and Discussion

### 3.1. Microstructures Evolution

#### 3.1.1. Microstructures and Phases

**Figure 1:**
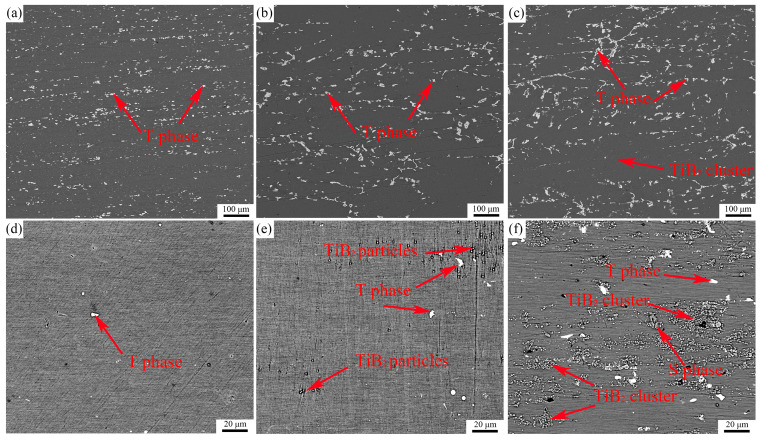
The microstructure scanning of the extruded (**a**–**c**) and aged composites (**d**–**f**) of the three composites.

**Figure 2:**
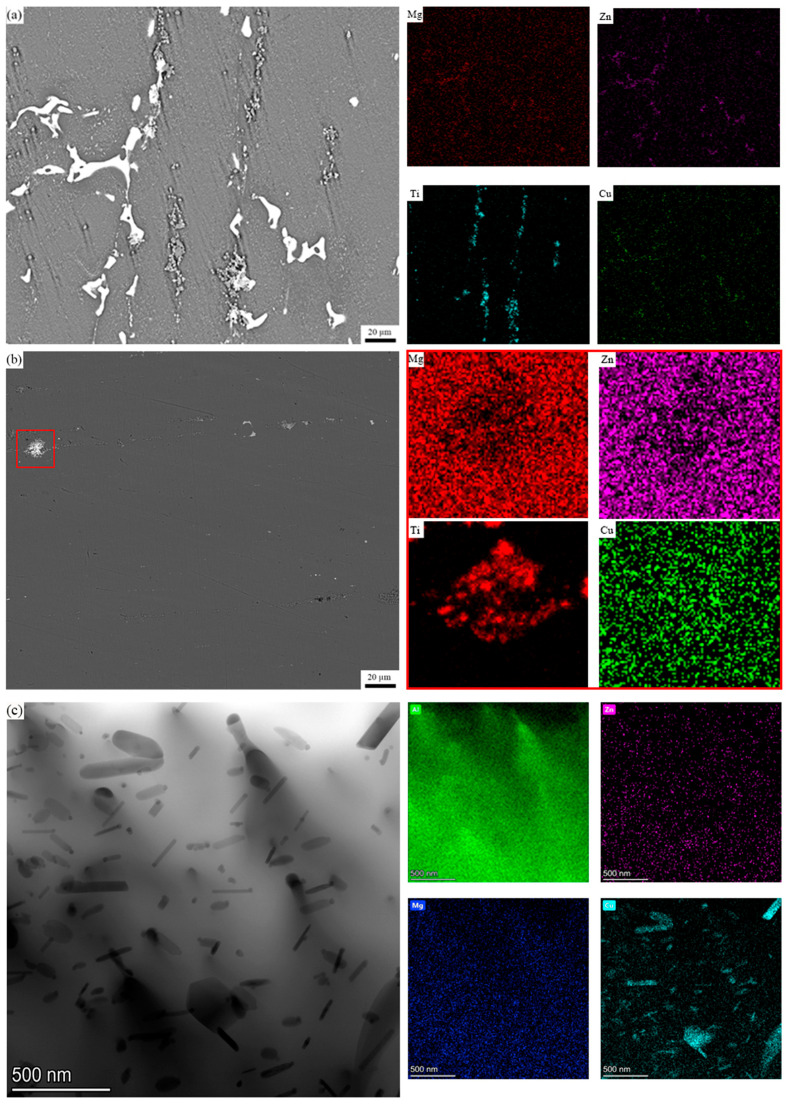
The extruded energy spectrum (EDS) picture of alloy B (**a**); solid solution energy spectrum (**b**,**c**).

Figure 1a–c show the scanning electron micrographs of the as-extruded microstructures of alloys A, B and C. The bright white area is the secondary phases left in the homogenization process or newly formed in the extrusion process, and the gray particles are the added TiB_2_ reinforcement particles. The phenomenon of dynamic precipitation of solute elements occurs during the hot extrusion process [33]. The precipitated secondary phases and some TiB_2_ particle clusters align in a fibrous manner along the deformation direction. According to the multi-temperature cross section of the Al-Zn-Mg-Cu-Zr phase diagram, the Al_2_CuMg (S) phase and the AlZnMgCu (T) phase can be retained in a relatively stable state for a long time at 450 °C [34]. The analysis of the DSC curve indicates that the precipitation temperature of the η-phase is below 220 °C. When the temperature exceeds 220 °C, the η-phase dissolves and exists in solid solution within the aluminum matrix. [35]. The precipitation temperature range of the S-phase is 250–400 °C. Therefore, the brittle second phases in the as-extruded alloy are a mixture of S phase and T phase [36]. Figure 2a shows the Energy Dispersive X-ray Spectroscopy (EDS) image of the as-extruded Alloy B, indicating that the T phase primarily consists of elements such as Al, Zn, Mg, and Cu. Figure 2a also reveals that a portion of the T phase precipitated and grows attached to the surfaces of the TiB_2_ particles.

**Figure 3:**
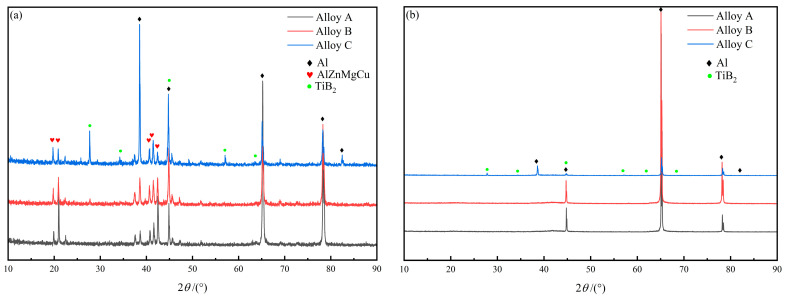
XRD curves of extruded (**a**) and aged composites (**b**).

Figure 3a,b present the XRD patterns of the three composites in the as-extruded and peak-aged states, respectively. The qualitative analysis of the composition types of secondary phases in alloys with different TiB_2_ particle contents was performed by examining the presence or absence of diffraction peaks in the XRD patterns. Figure 3a shows that when the content of TiB_2_ particles in the alloy continues to rise, the diffraction peaks of the mixture of T phase and S phase do not change significantly. It is observed that the TiB_2_ content has negligible influence on the precipitation behavior of secondary phases during extrusion. Figure 1d–f are the aging microstructure of the three composites. In alloys B and C, most of the sub-micron TiB_2_ particles are uniformly dispersed within the aluminum matrix. In Sample B, large TiB_2_ clusters are scarcely observed, whereas in Alloy C, significant clustering of TiB_2_ particles has formed. Furthermore, analysis indicates that the secondary phases in Composites A and B are almost completely dissolved back into the aluminum matrix after the solution heat treatment, while a portion of the secondary phases remains undissolved in Composite C. This is because the surrounding TiB_2_ particles hinder the re-dissolution of the secondary phases, which leads to the solution time of 3 wt.% TiB_2_ particle reinforced composites being longer, and the solution effect being the worst. The TiB_2_ particle content can also directly influence the volume fraction of secondary phases in the solution-treated alloy. As shown in Figure 3b, the diffraction peaks of the secondary phases in the XRD patterns disappeared after the heat treatment process. Furthermore, no distinct diffraction peaks of secondary phases are observed in the XRD curve of Alloy C. Figure 2b,c show the SEM and TEM images, along with the corresponding energy-dispersive X-ray spectroscopy (EDS) results, of Alloy B in the solution-treated state. It can be observed that elongated secondary phases rich in Cu elements remain within the aluminum matrix. This phenomenon is attributed to the low diffusion coefficient of Cu elements [37,38].

**Figure 4:**
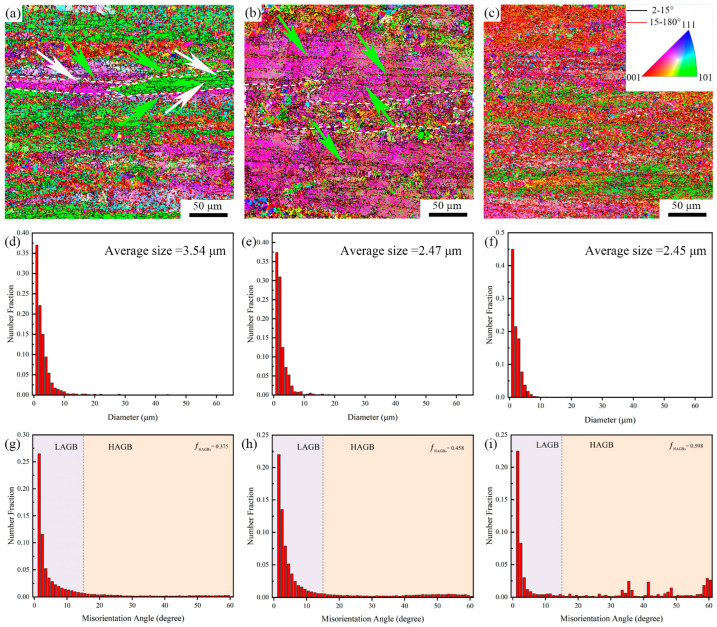
EBSD microstructure of the composites; statistics of grain size; grain boundary angle statistics; (**a**,**d**,**g**) Composite material A; (**b**,**e**,**h**) Composite material B; (**c**,**f**,**i**) Composites C.

Figure 4a–c present the EBSD microstructures of the three alloys in the as-extruded state, in which the red lines represent low-angle grain boundaries (misorientation angle between 2° and 15°), and the black lines represent high-angle grain boundaries (misorientation angle greater than 15°). It can be clearly seen from the diagram that the microstructure of the composite material presents a typical fibrous deformation structure. With the increase of TiB_2_ particle content, fine equiaxed recrystallized structure appears near the TiB_2_ particles, indicating that TiB_2_ particles promote the dynamic recrystallization of the composites during conventional extrusion deformation. In the as-extruded sample, the grain boundaries in the fibrous grains (as shown in the white dotted box in Figure 4a) (as shown in the black line in Figure 4a) are related to the formation of the subgrains structure [39]. In addition, the growth of subgrains changes the crystal orientation, and shows different crystal orientation relationships relative to the aluminum matrix of slender grains [39], as shown in the white arrows in Figure 4a. This is related to the continuous dynamic recrystallization mechanism of grains during deformation. In addition, as shown by the green arrow in Figure 4a, the serrated morphology at the grain boundary protrusion of the fibrous grains is considered to be the nucleation site of the discontinuous dynamic recrystallization (DDRX) grains. After the addition of TiB_2_ particles, the occurrence of subgrains coalescence produces finer continuous dynamic recrystallization (CDRX) equiaxed grain structure (as shown in the white dotted frame in Figure 4b). In addition, as shown by the green arrow in Figure 4b, the DDRX grain structure along the zigzag boundary develops into a chain structure with almost the same grain orientation. The CDRX and DDRX structures generated by extrusion deformation will play a positive role in the texture strengthening of the alloy, and then the work hardening rate will be improved. In addition, in alloys B and C, the CDRX and DDRX grain structures near TiB_2_ are subjected to higher extrusion strain [40]. Therefore, there is a higher dislocation density around the TiB_2_ particles. Therefore, according to the principle of precipitation kinetics, it can be inferred that the aging treatment process of alloy B and C should be faster than that of matrix alloy.

Figure 4d–f shows the grain size of the as-extruded composites with different TiB_2_ content. After extrusion deformation, the grain size of the three alloys decreased significantly. The average grain sizes of alloys A, B and C are 3.54 μm, 2.47 μm and 2.45 μm, respectively. This result fully shows that with the increase of TiB_2_ content, the grain size of the alloy will be significantly refined. This is because some TiB_2_ particles act as heterogeneous nucleation sites of α-Al matrix alloy during the solidification of composites. On the other hand, the existence of TiB_2_ particles hinders the diffusion of aluminum atoms and other atoms, thereby suppressing the growth of primary α-Al grains [41] and leading to grain refinement in the alloy.

Figure 4g–i is the grain boundary misorientation diagram of composites with different TiB_2_ particle content. It can be seen from Figure 4g–i, the matrix alloy exhibits a low-angle grain boundary (LAGB) fraction of 62.5% and a high-angle grain boundary (HAGB) fraction of 37.5%. With the addition of TiB_2_ particles, the LAGB fraction in the 1 wt.% TiB_2_ particle-reinforced aluminum matrix composite decreases to 54.2%, while the HAGB fraction increases correspondingly to 45.8%. When the TiB_2_ content reaches 3 wt.%, the LAGB fraction further decreases to 40.2%, and the HAGB fraction rises to 59.8%. This indicates that as the TiB_2_ particle content increases, the stored dislocation energy within the composite increases. This provides sufficient energy for the transformation of LAGBs into HAGBs, resulting in an increased proportion of dynamic recrystallization [42].

**Figure 5:**
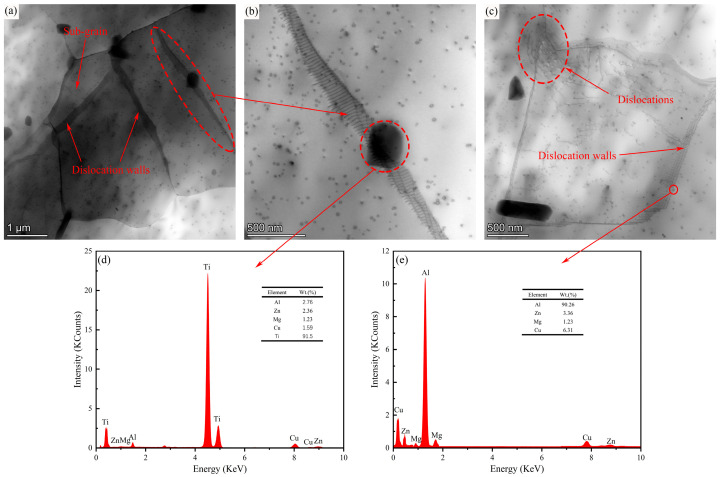
TEM microstructure of composite B (**a**–**c**) and the energy spectrum diffraction pattern at the corresponding position (**d**,**e**).

The substructure and precipitates in alloy B were observed and analyzed by transmission electron microscopy. The TEM micrograph of the B alloy after hot extrusion deformation is shown in Figure 5. Figure 5a shows that alloy B produces some sub-grains with a size of about 1 μm after extrusion deformation, and these sub-grains are surrounded by dislocation walls composed of parallel dislocations. The effect of TiB_2_ particles with a size of about 350 nm on dislocations is shown in Figure 5b. These particles will pin the dislocations during the dislocation movement. When the dislocation motion is blocked, the line tension forces the dislocation line to bend (twist) locally, forming geometrically necessary dislocations, thereby improving the strength of the material. During the deformation process, due to the precipitation conditions of some precipitated phases are satisfied, the precipitation phenomenon inevitably occurs [43]. It can be seen from Figure 5c that there are a large number of strain-induced precipitates, and the size of these precipitates is basically the same. These precipitated phases are due to the energy stored during the deformation process and some dislocations that can be used as nucleation sites are sufficient to exceed their nucleation barrier, which is conducive to the formation of precipitated phases [44]. Figure 5d,e show the EDX results of TiB_2_ particles and spherical precipitates in Figure 5b,c, which are represented by red circles. It can be seen that the main elements of TiB_2_ particles are Ti and B, and the main elements of the precipitated phase are Al, Cu, Zn and a small amount of Mg. These precipitates can play a role in pinning dislocations in grains and preventing grain growth and grain boundary collision. Meanwhile, the formation of precipitates also consumes the stored deformation energy, thereby reducing the driving force for recrystallization.

**Figure 6:**
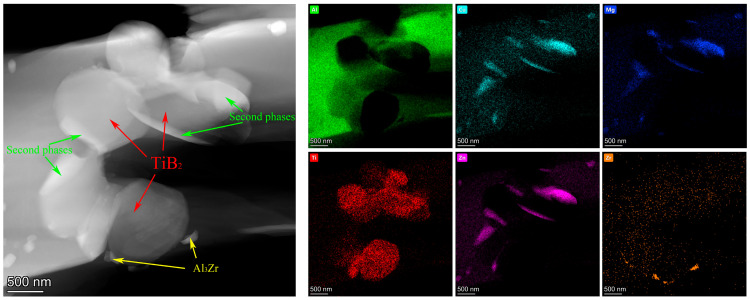
Local bright field TEM microstructure and energy spectrum of aged composite B.

Figure 6 shows a local bright-field TEM micrograph of the aged alloy B, in which some residual interfaces surrounded by localized TiB_2_ particles are observed. The interface phase adjacent to a single TiB_2_ particle was also observed. The energy spectra of Ti and solute elements are given. It can be inferred from the energy spectrum of various elements that these precipitated phases are mainly T phase, MgZn_2_ phase and Al_3_Zr phase. During the solution treatment process, local particle clusters prevent the dissolution of the interface. Therefore, after solution treatment, although most of the interfacial phases dissolve into the matrix, the aggregation of local TiB_2_ particles and the existence of grain boundaries affect the dissolution process of the interfacial phases.

**Figure 7:**
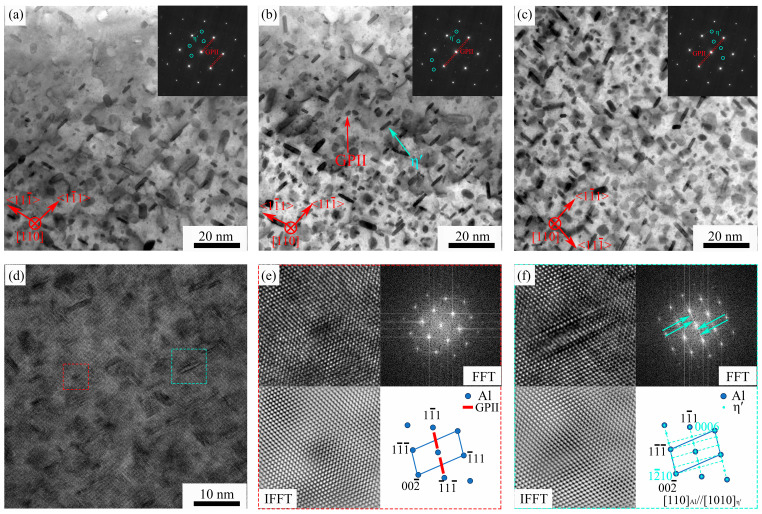
The bright field images of the precipitates (MPt) of the aged composites matrix and the corresponding selected area electron diffraction patterns (**a**–**c**), the high-resolution TEM (HRTEM) images of the composite B (**d**–**f**) and the Fourier transform (FFT), inverse Fourier transform (IFFT) and simulated electron diffraction images of the precipitates.

The primary strengthening precipitates in 7xxx series aluminum alloys during aging are GP zone, η′ and η phases [35,44,45]. These precipitates can be identified using Selected Area Electron Diffraction (SAED) patterns. Figure 7a–c presents bright-field transmission electron microscopy (BF-TEM) images of the matrix precipitates (MPt) and their corresponding SAED patterns along the <110>_Al_ zone axis for the three peak-aged alloys. By observing the images, it can be seen that a large number of tiny precipitates are distributed in the matrix of the three alloys, showing fine rod and oval shapes. Very weak diffraction stripes in the GP zone can be observed along the <111>_Al_ axis in the selected area electron diffraction pattern. The diffraction spots of η’ phase can be observed at 1/3{220} and 2/3{220}, and η’ phase can be observed in the peak-aged state of these three alloys. In the 7xxx series aluminum alloy, the GP zone is the precursor of the η’ phase, which generally presents a disk shape and precipitates along the {111}_Al_ surface. And the GP zone is completely coherent with the face-centered cubic (FCC) Al matrix. The size of the GP zone is generally small, generally about 2 nm. The η’ phase, with its relatively large size, exhibits a coherent or semi-coherent interface structure with the aluminum matrix. From the transmission images, it was not found that the number and size of the matrix precipitates of the three alloys were significantly different.

Figure 7d–f shows the high-resolution TEM (HRTEM) image of the alloy with 1 wt.% TiB_2_ content, along with the corresponding Fast Fourier Transform (FFT), Inverse Fast Fourier Transform (IFFT) patterns of the precipitates, and the simulated electron diffraction pattern. Two typical precipitates (outlined by red and green dotted lines) can be observed along the (1$\bar{1}$1)_Al_ plane. The distribution of diffraction spots along the [1$\bar{1}$1]_Al_ direction in the FFT pattern of the first phase confirms its identification as a GPII zone [37,46]. The GPII zone is fully coherent with the aluminum matrix, and its simulated lattice pattern is presented in Figure 7e. The FFT diffraction pattern of the second type of precipitate also shows diffraction spots distributed along the [1$\bar{1}$1]_Al_ direction, which can be confirmed as η′ phase by comparison. The η′ phase maintains a semi-coherent relationship with the matrix. Figure 7f presents the simulated pattern and the corresponding Inverse Fast Fourier Transform (IFFT) pattern. The orientation relationship between this η′ phase and the Al matrix is: [10$\bar{1}$0]_η′_//[110]_Al_; (0001)_η′_//(110)_Al_. Additionally, GPII zones can be observed as disc-shaped structures with diameters approximately ranging from 4 to 6 nm. The η’ phase has a width of about 5–7 nm and a length of about 8–10 nm. The two phases are not easy to distinguish in the thickness direction but have obvious differences in the plane size. Based on the coherence of the diffraction spots of the two phases, the two phases can be accurately distinguished in HR (high-resolution) micrographs. The precipitated phases in the other two alloys are the same as those of the alloy, so no other detailed explanation is made here.

#### 3.1.2. Effect of TiB_2_ on the Second Phase

During the solidification of an alloy, elemental segregation occurs, leading to the precipitation of a large amount of non-equilibrium eutectic phases at the grain boundaries [18,47]. Simultaneously, due to the interfacial energy at the solid–liquid interface, TiB_2_ reinforcement particles tend to form large-sized clusters at the grain boundaries. Moreover, within some of these coarse particle clusters, primary T phase is present, which reduces the contact area between the T phase and the aluminum matrix, thereby slowing down the dissolution rate of the T phase during the solution treatment process. According to Figure 1a–c, the as-extruded alloy exhibits a fibrous network structure of secondary phases composed predominantly of T phase with a small amount of S-phase. During solution treatment, the T phase gradually dissolves into the matrix, forming a solid solution. Elements at the interface preferentially dissolve into the aluminum matrix. As the solution treatment time increases, the interface between the T phase and the matrix gradually moves towards the center of the T phase, causing the T phase to spheroidize. The supersaturated solid solution of alloying elements formed near the interface also acts as a barrier, significantly reducing the dissolution rate of the T-phase. Furthermore, there are differences in the diffusion rates of the various alloying elements during the dissolution of the T phase. Zn diffuses into the matrix at the highest rate, followed by Mg, while Cu has the slowest diffusion rate. Consequently, with prolonged solution treatment time, the diffusion of Zn is the most extensive, leading to the partial transformation of some T phase into S phase [48]. The solidus temperature of the S phase ranges between 470 °C and 490 °C. At the solution temperature used in this experiment (475 °C), a small amount of S phase remains undissolved. When the alloy is subjected to stress, the residual S phase pins dislocations, causing significant dislocation pile-ups. This leads to inhomogeneous plastic deformation and stress concentration around the S phase. As the deformation increases, fine voids form, ultimately leading to fracture at the sites where these voids coalesce and grow. Additionally, the residual T phase, being brittle, readily initiates stress concentration and can cause secondary cracking, typically along high-angle grain boundaries [49,50,51]. Therefore, the residual T phase, S phase, and TiB_2_ clusters are common sites for crack initiation in the composite, adversely affecting both its tensile strength and elongation. In Alloy B, with the addition of a small amount of TiB_2_ (1 wt.%), effective nucleation sites are provided while TiB_2_ clustering is reduced. This improves the contact interface between the T-phase and the Al matrix, minimizes the formation of S phase, and ensures a high solubility of alloying elements. The trace amount of residual T phase and the absence of S phase transformation contribute to the enhancement of mechanical properties, particularly the elongation.

ZrAl_3_ with its high crystallization temperature tends to nucleate preferentially during alloy solidification, thereby providing additional nucleation sites for the aluminum matrix and promoting grain refinement. According to research by Xiao et al. [12,14], the precipitation temperature of the ZrAl_3_ phase exceeds 350 °C. During homogenization, the primary eutectic phases are largely eliminated; ZrAl_3_ particles can nucleate via both heterogeneous and (under specific conditions) homogeneous pathways, consistent with the literature on Zr-bearing dispersoids in aluminum alloys [52]. Therefore, in TiB_2_/Al–Zn–Mg–Cu composites, TiB_2_ particles can generally act as nucleation sites for the ZrAl_3_ phase at this stage, with this phenomenon being particularly pronounced near TiB_2_ clusters. However, agglomerated TiB_2_ particles and ZrAl_3_ phases also become sources of crack initiation during deformation, significantly impairing the material’s tensile strength and ductility. When an appropriate amount of TiB_2_ (1 wt.%) is added, the amount of coarse ZrAl_3_ phase can also be at an acceptable level. Furthermore, after extrusion deformation, ZrAl_3_ phases are fragmented, thereby minimizing their detrimental effects on the material’s tensile strength and ductility [49,52,53].

#### 3.1.3. Effect of TiB_2_ Particles on Precipitation Kinetics

The influence of TiB_2_ particle content on the precipitation kinetics can be analyzed from two aspects: its effect on solid solubility and the parameters of precipitation kinetics. Analysis of the precipitation kinetics model equation reveals that the content of solute elements in the alloy composition plays a decisive role in the volume fraction and size of the precipitate phases. During the solution heat treatment process, most of the secondary phases in the alloy dissolve into the aluminum matrix, resulting in a supersaturated solid solution after quenching. However, according to Figure 2b, a small amount of secondary phase, surrounded by TiB_2_ particle clusters and thus lacking sufficient contact with the matrix, fails to dissolve and remains in the alloy as residual T phase. Research by Geng et al. [20] indicates that the addition of reinforcement particles inevitably reduces the contact area between the secondary phases and the aluminum matrix. Consequently, these reinforcement particles lead to an effective concentration of solute elements in the alloy that is lower than the initial design value. The error introduced by the reinforcement particles has not yet been accurately quantified in current research. It is known that factors affecting this error mainly include the size and distribution state of the reinforcement particles. If the particles are dispersed uniformly within the alloy, the error value is smaller. However, when a large number of particle clusters form in the alloy, the diffusion speed and amount of some secondary phases into the aluminum matrix are reduced. Previous studies have shown that reinforcement particles influence precipitation kinetics through multiple mechanisms. While they accelerate the precipitation rate, they do not alter the sequence of precipitation phase formation. The difference in the coefficient of thermal expansion (ΔCET) between the aluminum matrix and the reinforcement particles leads to the formation of a high density of dislocations near the particles during quenching [54,55]. The presence of a substantial number of geometrically necessary dislocations (GNDs) near the reinforcement particles serves as the primary driving force for promoting the nucleation and rapid growth of precipitate phases. Taking Alloy B as an example, the density of geometrically necessary dislocations (GNDs) can be estimated using the following formula [56]:

(1) $$\rho_{G}=\frac{12V_{p}∆CET∆T}{(1-V_{p})bD}$$

*Vp* is the volume fraction of the reinforcement particles, *ΔCET* is the difference in the coefficient of thermal expansion between the particles and the matrix, b is the Burgers vector of the dislocations and D is the particle size (for Alloy B: *V_p_* = 0.6%; *CET_Al_* = 2.4 × 10^−5^ K^−1^, *CET_TiB_2__* = 7.8 × 10^−6^ K^−1^). In this study, high-quality TiB_2_ reinforcement particles at the micro-nano scale (200 nm–1 μm) were used. Assuming an average TiB_2_ particle diameter of 560 nm, the density of geometrically necessary dislocations (GNDs) generated upon quenching from the solution treatment temperature to room temperature is calculated to be 3.28 × 10^15^ m^−2^ [54,56]. By assuming that these dislocations are distributed within the matrix plastic deformation zone (MPDZ), Dutta et al. calculated the size of the MPDZ induced during quenching in an Al alloy reinforced with Al_2_O_3_ particles [57]:

(2) $$C_{c}={D\left(\frac{4\;*\;∆CET\;*\;∆T\;*\;E}{\left(5-4\nu \right)\sigma_{y}}\right)}^{1/2}$$

In the formula: *E* is Young’s modulus; *ν* is Poisson’s ratio; *σ_y_* is the yield strength. Calculations in this experiment show that the size of the MPDZ is 0.95D. This calculation model for the density of geometrically necessary dislocations (GNDs) generated by thermal mismatch during quenching and the size of the matrix plastic deformation zone (MPDZ) is based on an assumption: when the strain α caused by thermal mismatch exceeds the elastic strain limit of the material, screw dislocations are generated, which can propagate from the interface into the alloy matrix. Therefore, it can be inferred that a key factor affecting the size of the matrix plastic deformation zone (MPDZ) is the yield strength of the matrix alloy. The size of the MPDZ is inversely proportional to the yield strength. The calculation method for the thermal mismatch parameter α is: [54,55,56,57]:

(3) α=∆CET×∆T

In this study, when the composite was quenched from 475 °C to room temperature, the thermal mismatch strain (α) reached 0.0072. The maximum stress calculated by multiplying the thermal mismatch strain by Young’s modulus was 619.6 MPa, which may be insufficient to promote the formation of screw dislocations. However, due to the varying shapes of TiB_2_ particles, the distribution of elastic strain energy around the particles also changes, potentially leading to the formation of precipitate-free zones (PFZs) near the TiB_2_ particles [58]. In this work, the addition of TiB_2_ reinforcement particles did not alter the model parameters related to the diffusion coefficients of solute elements.

### 3.2. Orientation Relationship

#### 3.2.1. The Interface Relationship Between TiB_2_ Particles and Al-Matrix

**Figure 8:**
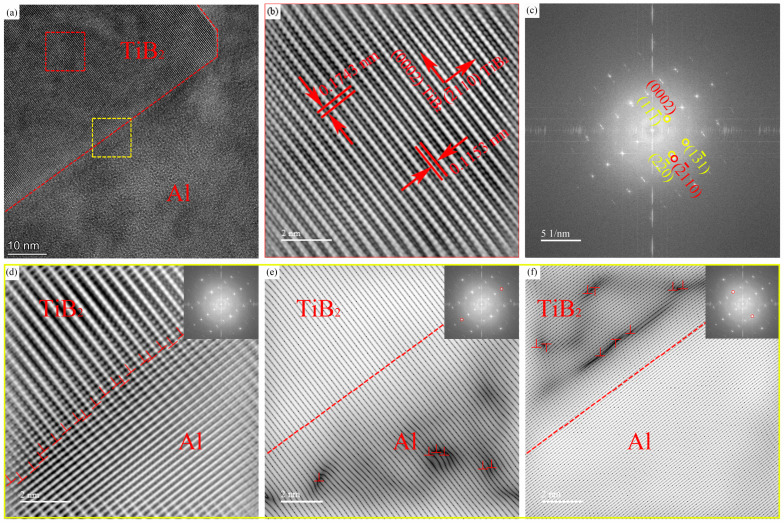
The composite B high-resolution images, Fourier images and inverse Fourier transform images of the interface between TiB_2_ reinforcement particles and alloy matrix. (**a**) HRTEM micrograph; (**b**) corresponding iFFT image of the TiB_2_ particle and the measured interplanar spacing; (**c**) FFT diffraction pattern between TiB_2_ particles and Al matrix. (**d**–**f**) iFFT analysis about the interfacial dislocations at the interface between TiB2 particle and Al matrix as marked by yellow rectangle in (**a**).

Figure 8a shows a high-resolution micrograph of the interface between the TiB_2_ reinforcement particles and the alloy matrix in the composite material. The experiments were conducted along the [112]_Al_ zone axis. Figure 8b,c show the magnified view of the region marked by the red box in Figure 8a and its corresponding Fast Fourier Transform (FFT) pattern, respectively. Analysis of the HRTEM image and the corresponding Fast Fourier Transform (FFT) pattern confirmed the crystallographic orientation relationship between the aluminum matrix and the TiB_2_ particle as: [112]_Al_//[01-10]_TiB_2__ and (11$\bar{1}$)_Al_//(0002)_TiB_2__. The measured interplanar spacings for the (0002) and ($\bar{2}$110) planes of the TiB_2_ particle were 0.1743 nm and 0.1153 nm, respectively. These values are in close agreement with the theoretical interplanar spacings for these planes, which are 0.1614 nm and 0.1215 nm. Inverse Fast Fourier Transform (IFFT) images of the TiB_2_/Al interface region Figure 8d–f reveal the presence of dislocations at the interface. Based on this analysis, it is concluded that the interface between the TiB_2_ particle and the aluminum matrix has a semi-coherent structure.

#### 3.2.2. The Interface Relationship Between TiB_2_ Particles and MgZn_2_ Phase

**Figure 9:**
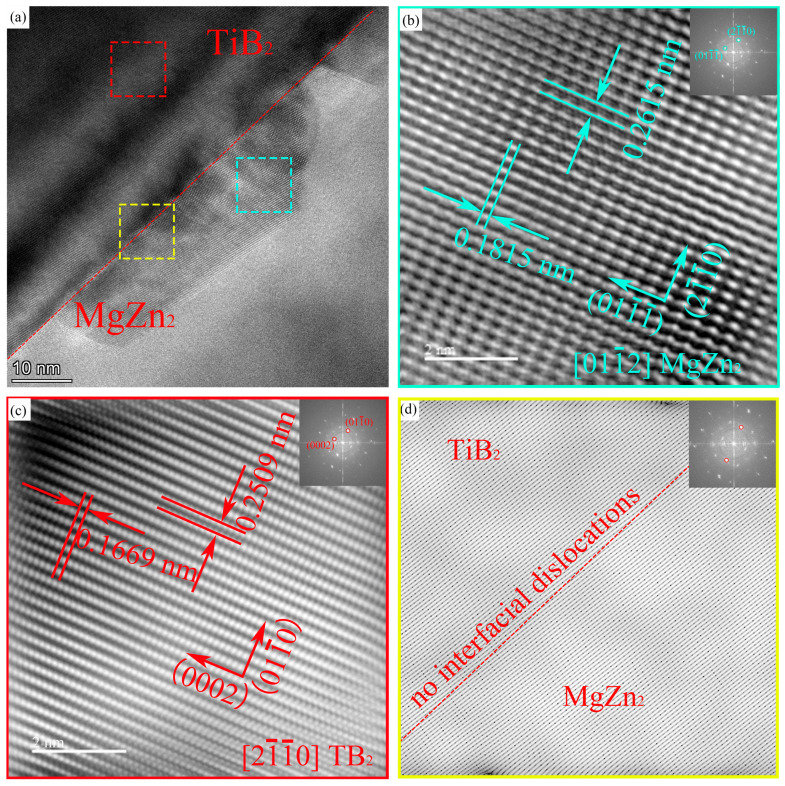
The Composite B HRTEM micrographs of the interface between TiB_2_ particles and MgZn_2_ phase; Fourier images and inverse Fourier transform images. (**a**) HRTEM micrograph; (**b**) and (**c**) corresponding IFFT patterns of the TiB2 particle and the MgZn_2_ phase with their FFT diffractograms (insets), respectively; (**d**) the interface structure analysis about the TiB_2_/MgZn_2_ interface.

Figure 9 shows an HRTEM micrograph of the interface between a TiB_2_ particle and an MgZn_2_ (η) phase. Analysis of the magnified region of the η phase and the corresponding FFT pattern (Figure 9b) indicates that the incident beam direction is close to the [01$\bar{1}$2]_MgZn_2__ zone axis. The measured interplanar spacings for the (01$\bar{1}\bar{1}$) and (2$\bar{1}\bar{1}$0) planes are 0.2615 nm and 0.1815 nm, respectively.

Analysis of the magnified region of the TiB_2_ particle and its corresponding FFT pattern Figure 9c shows that the incident beam direction is close to the [2$\bar{1}\bar{1}$0]_TiB_2__ zone axis. The measured interplanar spacings for the (0002) and (01$\bar{1}$0) planes are 0.1669 nm and 0.2509 nm, respectively.

By comparing Figure 9b,c, along with their corresponding FFT patterns, the orientation relationship between the η phase and the TiB_2_ particle is determined to be: [01$\bar{1}$2]_η_//[2$\bar{1}\bar{1}$0]_TiB_2__, (01$\bar{1}\bar{1}$)_η_//(01$\bar{1}$0)_TiB_2__, (2$\bar{1}\bar{1}$0)_η_//(0002)_TiB_2__. Furthermore, analysis of the IFFT image Figure 9d of the interface between the TiB_2_ particle and the η phase (marked by the yellow dashed line) reveals no significant interfacial dislocations. Therefore, it can be inferred that the interface between the η phase and the TiB_2_ particle is fully coherent.

### 3.3. Mechanical Properties

#### 3.3.1. Hardness and Tensile Properties

**Figure 10:**
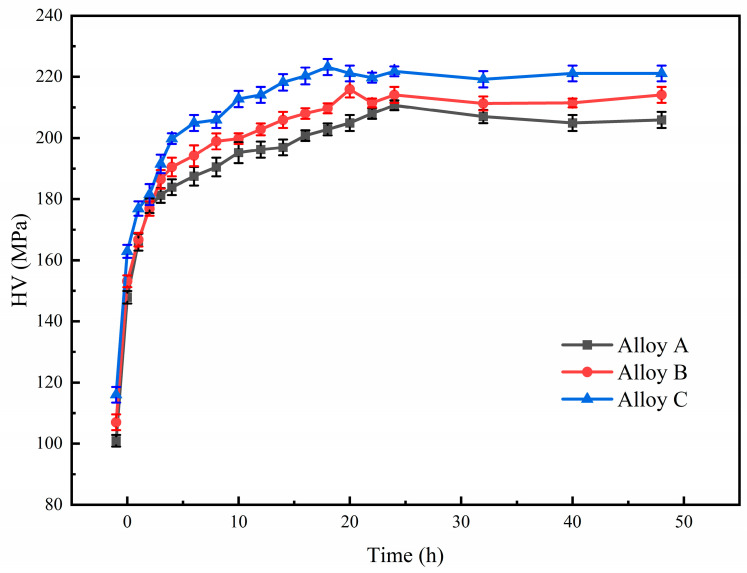
Peak age hardening curve of composite at 120 °C.

Figure 10 shows the peak aging hardening curves of the three alloys at 120 °C. As age-hardening alloys, all three alloys show rapid hardening response with the increase in aging time. As the content of TiB_2_ particles increases in the alloy, the hardness of the as-extruded composites also shows an increasing trend, with values of 101 HV, 107 HV, and 116 HV, respectively. The hardness of the three samples after solution treatment is 148 HV, 153 HV and 163 HV, respectively. The contribution of solid solution strengthening to the alloy’s strength is positively correlated with the solute content [59]. Given that the solute contents of the three alloys are nearly identical, their hardness remains in a state of dynamic equilibrium during the solution treatment process. The peak aging of A, B and C alloys is 24 h, 20 h and 18 h, respectively, and the hardness of peak aging is 209 HV, 216 HV and 223 HV, respectively. After the hardness of the three alloys rapidly reaches the peak, and with the extension of aging time, the hardness basically does not fluctuate greatly. In addition, it was observed that the response speed of aging hardening gradually accelerated with the increase of TiB_2_ content. Therefore, an increase in TiB_2_ particle content not only enhances the peak hardness of the composite but also accelerates its age-hardening kinetics.

#### 3.3.2. Tensile Properties and Work Hardening Rate Curves

**Figure 11:**
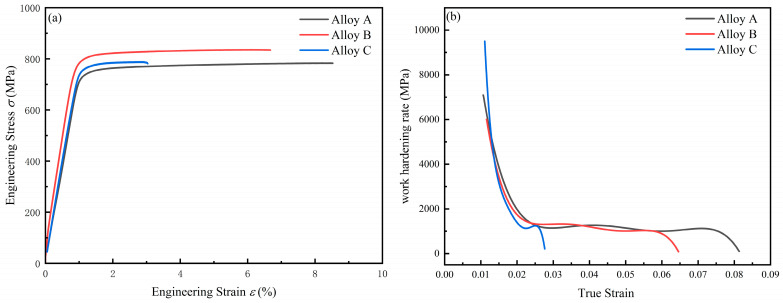
(**a**) Engineering stress–strain curve of peak-aged composite at room temperature; (**b**) curve of the relationship between work hardening rate and plastic deformation stress.

Figure 11 is the room temperature engineering stress–strain curves of peak-aged three kinds of composites. When the content of TiB_2_ is 0, the yield strength of the alloy is 743 MPa, the tensile strength is 781 MPa, and the elongation is 8.5%. When the content of TiB_2_ is 1%, the yield strength of the alloy is 801 MPa, the tensile strength is 831 MPa, and the elongation is 6.7%. When the content of TiB_2_ is 3%, the yield strength of the alloy is 759 MPa, the tensile strength is 788 MPa, and the elongation is 2.9%. The tensile strength of the composite material generally exhibits a trend of first increasing and then decreasing with the continuous increase in TiB_2_ reinforcement particle content. This is because when 1% TiB_2_ is added, TiB_2_ particles act as heterogeneous nucleation cores and play a role in refining the matrix grains. TiB_2_ particles have a pinning effect on dislocation movement and the dispersion distribution of TiB_2_ particles has a uniform strengthening effect on the matrix. When 3% TiB_2_ is added, too many particles form a large number of particle clusters during solidification. These clusters are not conducive to the re-dissolution of the second phase, and these clusters cause stress concentration, so the elongation is also greatly reduced. Additionally, Table 2 presents the strain hardening exponents (n-values) corresponding to the true stress–strain curves of the three alloys, which are described by the Hollomon equation: [60]:

(4) $$\sigma ={K\varepsilon }_{p}^{n}$$

*σ*: True stress; *ε_p_*: true strain; K: strength coefficient; *n*: the strain hardening index. Analysis reveals that the strain hardening exponent decreases with increasing TiB_2_ particle content. Figure 11b shows the relationship between the strain hardening rate and true plastic deformation stress for the three composites. In the initial stage of plastic deformation, the strain hardening rates of all three composites are relatively high, and the initial strain hardening rate increases with higher TiB_2_ content. As plastic deformation progresses, the strain hardening rate first decreases rapidly and then stabilizes. Composite C exhibits the most pronounced decay in strain hardening rate, with the steepest absolute slope, followed by Composite B, while the matrix alloy shows the smallest absolute slope.

**Figure 12:**
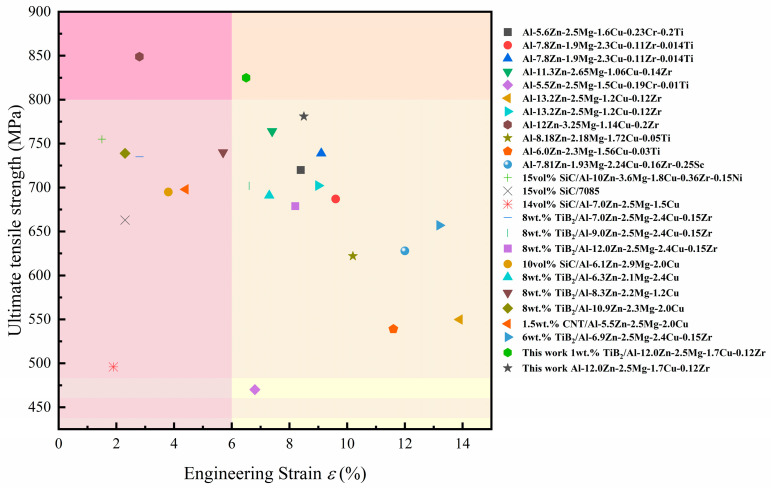
Summary of tensile properties of high strength aluminum alloy in this work and related research [5,18,46,61,62,63,64].

Consequently, the composite with 1 wt.% TiB_2_ content possesses well-balanced strain hardening characteristics, achieving high strength while maintaining good uniform plastic deformation capability. Compared with previous studies, 1 wt.% TiB_2_/Al-12Zn-Mg-Cu-Zr composites have excellent strength and plasticity balance (Figure 12). In the process of extrusion deformation, the phenomenon of uneven deformation between TiB_2_ and Al leads to a large number of geometrically necessary dislocations (GNDs). A large number of slip dislocations are gathered at the TiB_2_ interface, resulting in stress concentration during continuous deformation. Some studies suggest that Al_3_Zr can be used as a buffer layer for dislocations. A large number of misfit dislocations at the interface absorb slip GND, thereby slowing down local stress. Furthermore, TiB_2_ particles can effectively promote nucleation by serving as heterogeneous nucleation sites within the composite material, while also restricting grain growth by impeding the movement of grain boundaries. The fine grains surrounding the TiB_2_ particles provide multiple slip systems and grain boundaries for plastic deformation, which helps to absorb dislocations and reduce stress concentration. Therefore, the composite with 1 wt.% TiB_2_ can have excellent comprehensive mechanical properties.

#### 3.3.3. Strengthening Mechanism

The superior tensile properties of the TiB_2_/Al-Zn-Mg-Cu-Zr composite are primarily attributed to its refined matrix microstructure and the effective strengthening contribution of TiB_2_ particles. After deformation, the composite exhibits grain refinement, a higher density of dislocations, and a more uniform distribution of TiB_2_ particles, consequently leading to superior mechanical properties. Taking 1 wt.% TiB_2_/Al-12Zn-2.2Mg-1.7Cu composite as an example, the contribution of its yield strength is mainly composed of the following aspects: (1) grain boundary strengthening; (2) Solid solution strengthening; (3) Dislocation strengthening; (4) Reinforcement particle load transfer strengthening; (5) Precipitated phase strengthening. The following formula describes the yield strength of the material using a multi-mode strengthening model [65]:

σ_YS_ = σ_0_ + σ_gb_ + σ_p_ + σ_s_ + σ_d_ (5)

where σ_0_ represents the driving force required for dislocation glide within the grains of pure aluminum during tensile deformation (about 10 MPa).

Integrated analysis of EBSD maps and mechanical properties reveals that a decrease in grain size leads to an increase in the number of grain boundaries, thereby improving the overall strength of the alloy. During material deformation, grain boundaries hinder the motion of dislocations within a grain or across adjacent grains, which contributes positively to the yield strength of the alloy. The contribution of grain boundary strengthening can be calculated using the Hall-Petch equation [32]:

σ_gb_ = K_y_d^−1/2^ (6)

In the equation, d represents the average grain diameter, and K_y_ represents a constant reflecting the influence of grain boundaries on strength (for 7xxx series alloys, K_y_ = 0.12 MPa/m^1/2^). The contribution of grain boundary strengthening to the yield strength of the as-extruded composite is 38.34 MPa.

When the particle size is less than 1 μm, the Orowan strengthening effect plays a dominant role. The pinning effect of micron-sized TiB_2_ particles on dislocations forces them to bypass the particles and form Orowan dislocation loops. The factors influencing this strengthening effect include the particle size and dispersion degree, with a higher dispersion degree leading to a better strengthening outcome. Specifically, it can be expressed by the following formula [49]:

(7) $$\sigma_{p-{TiB}_{2}}=0.127M\;*\;\ln\frac{d}{b}\;*\;\frac{Gb}{\lambda \sqrt{1-\nu }}$$

In the formula, *M* represents the Taylor factor (its value is 2.2), *G* represents the shear modulus (the shear modulus of the face-centered cubic 7xxx series aluminum alloy is 27.80 GPa), *b* represents the value of the Burgers vector (0.286 nm), and *v* represents the Poisson’s ratio (its value is 0.33). *λ* represents the particle spacing, and the calculation method is as follows [49]:

(8) $$\lambda =\frac{d}{2}\times {\left(\frac{2\pi }{{3ƒ}_{v}}\right)}^{0.5}$$

In the formula, *d* represents the average diameter of the particles, and ƒ*_v_* represents the volume fraction of the particles. The average size of TiB_2_ particles is about 0.56 μm, and its volume fraction v is 0.6% by mass percentage conversion [32]. The strengthening effect of particles is about 15.13 MPa.

The main influencing factor of the precipitation strengthening mechanism is the size of the precipitated phase: the small-sized precipitates (such as atomic clusters) in the early stage of aging are mainly dislocation cutting mechanism, while the larger-sized precipitates are difficult to shear due to dislocations. It will force dislocations to choose to bypass the mechanism and form an Orowan dislocation ring, which can be explained by the Orowan mechanism [32]. According to the transmission electron microscopy (TEM) analysis, the main precipitated strengthening phases in the peak-aged matrix are η′ phase. Kocks statistical equation is an effective way to calculate the strengthening of aging precipitates [66]:

(9) $$\sigma_{{p-\eta }^{\prime }}=\frac{0.4MGb}{\pi \sqrt{1-v}}\times \frac{\ln\left(d\frac{\sqrt{\frac{2}{3}}}{b}\right)}{\sqrt{\frac{2}{3}}d\left(\sqrt{\frac{\pi }{4V}}-1\right)}$$

The average size of the aging precipitates in the alloy is approximately 7.1 nm. According to the 3DAP (Three-Dimensional Atom Probe) test results from Wang et al. [26]., the volume fraction of the precipitates in the T6-treated 7xxx series alloy is about 1.7%. The calculated contribution of precipitation strengthening is approximately 411.62 MPa.

Solid solution strengthening is primarily achieved by the dissolution of alloying elements into the matrix to form a solid solution. The difference in atomic radius between various solute elements and aluminum atoms varies (R_Al_ = 0.143 nm, R_Zn_ = 0.134 nm, R_Mg_ = 0.162 nm, R_Cu_ = 0.128 nm), leading to differences in the strength enhancement provided by different solid solutions. The magnitude of solid solution strengthening can be calculated using Equation (10):

(10) $$\sigma_{s}=\sum k_{j}c_{j}^{m}$$

In the formula, *K_j_* is the strengthening factor of the element (*k_Zn_* = 2.90 MPa/wt%, k_Mg_ = 18.60 MPa/wt%, *k_Cu_* = 13.80 MPa/wt%), and m is 1 [32]. The value of solid solution strengthening is about 99.18 MPa.

The deformation ability of various phases in TiB_2_/Al-Zn-Mg-Cu composites is different, so there is a gradient deformation phenomenon in the material during plastic deformation. In order to make the gradient deformation reach a relative equilibrium state, a large number of geometrically necessary dislocations (GNDs) will be generated around the particles. However, TiB_2_ particles and aluminum have different thermal expansion coefficients, and the composites still have dislocations after heat treatment. In addition, TiB_2_ particles can promote dislocation proliferation and offset some of the dislocations that disappear during heat treatment. Therefore, some GNDs in the composites are retained, and the strength is improved. The contribution of dislocation density increase to strength can be calculated by dislocation strengthening equation [49] (Equation (11)).

(11) $$\sigma_{d}=M\alpha Gb\sqrt{\rho }$$

In the equation, *M* represents the average Taylor orientation factor (taken as 3.05), α is a constant (for FCC metals, α = 0.2), and ρ denotes the dislocation density (which can be calculated using Equation (1)). As provided earlier, the density of geometrically necessary dislocations (GNDs) in Composite B is 3.28 × 10^15^ m^−2^. Consequently, the calculated contribution of dislocation strengthening is 278.91 MPa.

Therefore, the contributions of grain boundaries, TiB_2_ reinforcement particles, aging precipitates, solid solution strengthening, and dislocation strengthening to the yield strength are 38.34 MPa, 15.13 MPa, 411.62 MPa, 99.18 MPa, and 278.91 MPa, respectively. Evidently, under T6 heat treatment conditions, the enhancement of the yield strength in the as-extruded composite is primarily attributed to the contributions from precipitates and dislocations.

## 4. Conclusions

In summary, this experimental work investigated the microstructure, hardness, and tensile properties of TiB_2_/Al-Zn-Mg-Cu composites after hot extrusion deformation and T6 heat treatment. The research methods primarily included X-ray diffraction (XRD), transmission electron microscopy (TEM), scanning electron microscopy (SEM), and tensile testing. The conclusions are as follows:

(1) The in situ TiB_2_/Al-12Zn-2.2Mg-1.7Cu composite with 1 wt.% TiB_2_ content after extrusion deformation and T6 heat treatment has a tensile strength of 831 ± 5 MPa and an elongation of 6.7%. Compared with the matrix alloy, the tensile strength is increased by 5.3%, while the elongation is only slightly decreased. After adding an appropriate amount of TiB_2_, the mechanical properties of the alloy have better strength and plasticity. Therefore, this study determined the better addition amount of TiB_2_ particles in Al-12Zn-2.2Mg-1.7Cu alloy, which can provide a reference for subsequent composite research.

(2) The average grain sizes of the as-extruded matrix alloy and the TiB_2_/Al-12Zn-2.2Mg-1.7Cu composites with TiB_2_ contents of 1 wt.% and 3 wt.% are 3.54 μm, 2.47 μm, and 2.45 μm, respectively. This demonstrates that TiB_2_ reinforcement particles can effectively refine the grains. The refinement mechanism operates through two primary aspects: firstly, under the alloy system and processing conditions of this study, the nucleation-promoting effect of TiB_2_ particles has been further quantified; secondly, these particles effectively hinder the diffusion of aluminum and solute atoms, thereby restricting the growth of the primary grains, ultimately leading to grain refinement in the composite. With the gradual increase of TiB_2_ content, the effect of solid solution treatment is getting worse and worse. This is because TiB_2_ particles reduce the contact area between the second phase and the aluminum matrix, thus slowing down the dissolution rate of solute atoms. Excessive residue of the second phase also has a serious negative impact on the mechanical properties of the composites. Furthermore, as the TiB_2_ particle content increases, the stored dislocation energy within the composite rises. This provides sufficient driving force for the transformation of low-angle grain boundaries (LAGBs) into high-angle grain boundaries (HAGBs), consequently leading to a gradual increase in the proportion of dynamic recrystallization.

(3) The main precipitated phase of the composites with 1 wt.% TiB_2_ content in T6 state is GPII zone and η′. The orientation relationship between η′ phase and Al matrix is: [10$\bar{1}$0]_η′_//[110]_Al_; (0001)_η′_//(110)_Al_. The lattice orientation relationship between Al matrix and TiB_2_ is [112]_Al_//[01$\bar{1}$0]_TiB_2__, (11$\bar{1}$)_Al_//(0002)_TiB_2__. The orientation relationship between MgZn_2_ phase and TiB_2_ particles can be determined as [01$\bar{1}$2]_MgZn_2__//[2$\bar{1}\bar{1}$0]_TiB_2__, (01$\bar{1}\bar{1}$)_MgZn_2__//(01$\bar{1}$0)_TiB_2__, (2$\bar{1}\bar{1}$0)_MgZn_2__//(0002)_TiB_2__. No obvious interface dislocation was observed at the interface between MgZn_2_ phase and TiB_2_, and there was a good coherent relationship.

(4) A multi-component strengthening model for the composite was established to quantitatively assess the contributions of various strengthening mechanisms. The strengthening contribution of various strengthening mechanisms in the composites was quantified. Finally, the research shows that the main strengthening methods of the composites in the peak aging state include precipitation phase strengthening and dislocation value-added strengthening.

## Figures and Tables

**Table 1:** Chemical composition elements of TiB_2_/Al-Zn-Mg-Cu composites (wt.%).

Alloy	Zn (wt.%)	Mg (wt.%)	Cu (wt.%)	Zr (wt.%)	Ti (wt.%)	B (wt.%)	Al (wt.%)
A	12.29	2.72	1.75	0.112	0	0	Bal.
B	12.69	2.81	1.69	0.102	0.48	0.4	Bal.
C	12.35	2.69	1.68	0.121	1.61	0.82	Bal.

**Table 2:** Mechanical properties of peak-aged TiB_2_/Al-Zn-Mg-Cu composites.

Alloy	σ_0.2_ (MPa)	σ_b_ (MPa)	At (%)	E (GPa)	n
A	743 ± 2	781 ± 3	8.5 ± 0.5	74 ± 1	0.081
B	801 ± 3	831 ± 5	6.7 ± 0.3	75 ± 2	0.058
C	759 ± 2	788 ± 4	2.9 ± 0.4	78 ± 2	0.044

## Data Availability

The original contributions presented in this study are included in the article. Further inquiries can be directed to the corresponding author.
